# A multicenter phase II study of the combination of oxaliplatin, irinotecan and capecitabine in the first-line treatment of metastatic colorectal cancer

**DOI:** 10.1038/sj.bjc.6605075

**Published:** 2009-05-12

**Authors:** E Vasile, G Masi, L Fornaro, S Cupini, F Loupakis, S Bursi, I Petrini, S Di Donato, I M Brunetti, S Ricci, A Antonuzzo, S Chiara, D Amoroso, M Andreuccetti, A Falcone

**Affiliations:** 1 UO Oncologia Medica, Azienda USL 6, Istituto Toscano Tumori, Viale Alfieri 36, Livorno 57100, Italy; 2 UO Oncologia Medica, Azienda Ospedaliera Pisana, Istituto Toscano Tumori, Via Roma 55, Pisa 56100, Italy; 3 Sez. Oncologia Medica, Azienda USL 6, Piombino (LI), Istituto Toscano Tumori, Italy; 4 UO Oncologia Medica, Istituto Tumori IST, Genova; Italy; 5 UO Oncologia Medica, Ospedale della Versilia, Istituto Toscano Tumori, Lido di Camaiore (LU), Italy; 6 Dipartimento di Oncologia, dei Trapianti e delle Nuove Tecnologie in Medicina, Università degli studi di Pisa, Via Roma 55, Pisa 56100, Italy

**Keywords:** first-line treatment, metastatic colorectal cancer, triple drug combination, XELOXIRI

## Abstract

The triple drug combination consisting of irinotecan, oxaliplatin and 5-fluorouracil (FOLFOXIRI) has demonstrated higher activity and efficacy compared to the doublet FOLFIRI. 5-Fluorouracil could be substituted in FOLFOXIRI regimen by capecitabine, an oral fluoropyrimidine with similar efficacy. Recently, a dose-finding trial has demonstrated the feasibility of the combination of irinotecan, oxaliplatin and capecitabine (XELOXIRI) and established their recommended doses. The aim of this study was to evaluate the activity of XELOXIRI. A total of 36 patients with unresectable metastatic colorectal cancer received irinotecan 165 mg m^−2^ and oxaliplatin 85 mg m^−2^ on day 1 plus capecitabine 2000 mg m^−2^ per day orally in two doses from day 1 to day 7, every 2 weeks. Grade 3–4 toxicities were infrequent, expect for neutropenia and diarrhoea, which were each observed in 30% of patients. Two complete and twenty-two partial responses were obtained, corresponding to an overall response rate of 67% (95% CI 51.4–82%). After a median follow-up of 17.7 months, the median progression-free and overall survival were 10.1 and 17.9 months, respectively.

The substitution of 5-fluorouracil with capecitabine, in combination with irinotecan and oxaliplatin, is feasible and does not impair the activity of the regimen. However, the XELOXIRI combination is associated with a high incidence of diarrhoea and, therefore, should be considered as a not preferable alternative to FOLFOXIRI.

In the past decade, the advent of oxaliplatin and irinotecan has led to changes in the first-line treatment of metastatic colorectal cancer (mCRC; Saunders and Iveson, 2006). In fact, the combination of one of these new cytotoxic drugs with 5-fluorouracil (5-FU) and leucovorin (LV) significantly increases tumour response and prolongs survival of patients with unresectable advanced colorectal cancer over 5-FU/LV alone (Punt, 2004). Moreover, a pooled analysis of seven phase III trials comparing 5-FU/LV plus irinotecan or oxaliplatin containing doublets *vs* 5-FU alone demonstrated that survival of mCRC patients might be improved administrating all the three active drugs in the course of the disease. However, in a sequential strategy, 20–50% of patients who progress after first-line chemotherapy cannot receive second-line treatment, mainly because of deterioration of their performance status and liver function (Grothey *et al*, 2004). Furthermore, another pooled analysis indicated that there is a strong correlation between the response rate to first-line chemotherapy and the possibility of a postchemotherapy radical resection of metastases that may be associated with long-term survival (Folprecht *et al*, 2005).

Keeping these concepts in mind, the GONO group developed in phases I and II trials a triple-drug combination of oxaliplatin, irinotecan and 5-FU/LV named FOLFOXIRI (Falcone *et al*, 2002; Masi *et al*, 2004) and compared this combination to a standard doublet combination of 5-FU/LV plus irinotecan (FOLFIRI) in a phase III study on 244 mCRC patients (Falcone *et al*, 2007). The treatment with first-line FOLFOXIRI was feasible, associated with manageable toxicities and obtained a higher tumour response rate and a higher postchemotherapy radical resection of metastases rate. FOLFOXIRI also significantly increased progression-free survival (PFS) and overall survival over FOLFIRI.

For its activity, the triple-drug combination should be preferred especially if response rate is the major goal of treatment, whereas in other situations sequential therapy could be a useful alternative (Koopman *et al*, 2007).

Capecitabine is an oral fluoropyrimidine prodrug that achieves tumour-selective generation of 5-FU through conversion by the thymidine phosporylase enzyme that is more active in CRC cells compared with healthy tissue (Walko and Lindley, 2005). Different phase III trials have shown that capecitabine is at least as active and effective as 5-FU in the first-line treatment of mCRC, with a superior safety profile (Hoff *et al*, 2001; Van Cutsem *et al*, 2001; Cassidy *et al*, 2002). Moreover, the use of capecitabine instead of 5-FU, either with irinotecan or oxaliplatin, confirmed the activity and efficacy of the drug (Cassidy *et al*, 2004; Koopman *et al*, 2007). Based on these results, the triple combination of capecitabine with oxaliplatin and irinotecan appears to be an interesting regimen to be studied in mCRC patients that could simplify the treatment delivery and reduce the complications related to the central venous catheter compared to infusional 5-FU, as used in the FOLFOXIRI regimen.

Different schedules of capecitabine emerged from phase I trials (Budman *et al*, 1998; Mackean *et al*, 1998). It is worth noting that mathematical methods applied to the definition of the ideal treatment schedule suggested that the optimal duration of treatment with capecitabine is 7 days and predicted that drug delivery beyond 7 days could contribute to toxicity, with diminishing anticancer benefit (Traina *et al*, 2008). Moreover, a randomised phase II trial conducted by Scheithauer *et al*. demonstrated that a dose-intensified bimonthly combination of oxaliplatin plus capecitabine administered for 7 days followed by 7 days of rest is as safe and feasible as the combination of oxaliplatin on day 1 with capecitabine administered from day 1 to day 14 every 3 weeks, with higher RR and PFS for the bimonthly regimen (Scheithauer *et al*, 2003).

On these bases, the GONO performed a phase I trial in mCRC patients to establish the recommended dose of capecitabine in combination with fixed doses of irinotecan and oxaliplatin (XELOXIRI), administered at the same doses of the GONO-FOLFOXIRI regimen (Fornaro *et al*, 2009). The study demonstrated the feasibility of XELOXIRI at the recommended dose of capecitabine 2.000 mg m^−2^ per day on days 1–7 in combination with oxaliplatin 85 mg m^−2^ and irinotecan 165 mg m^−2^ on day 1, repeated every 2 weeks, but has also pointed out a large interpatient variability on the tolerance and on the pharmacokinetic values of the drugs. For these reasons, we decided that XELOXIRI should be evaluated in a phase II trial on a larger number of patients. In this study, we report the results of the phase II study with the XELOXIRI combination as the first-line treatment of unresectable metastatic colorectal cancer patients.

## Materials and methods

### Patients selection

Main eligibility criteria included histologically confirmed diagnosis of colorectal adenocarcinoma with unresectable metastatic disease; measurable disease according to Response Evaluation Criteria in Solid Tumors (RECIST) criteria; age 18–75 years; ECOG performance status (PS) <2 for patients aged ⩽70 years and ECOG PS=0 for patients aged >70 years; adequate bone marrow reserve (leukocyte count ⩾3.500 per mm^3^, neutrophil count >1.500 per mm^3^, platelet count ⩾100.000 per mm^3^); adequate kidney and liver functions (serum creatinine ⩽1.3 mg per 100 ml, serum bilirubin <1.5 mg per 100 ml, and aspartate aminotransferase, alanine aminotransferase and alkaline phosphatase <2.5 × upper normal values (<5 × upper normal values if liver metastases were present)). Previous fluoropyrimidine-based adjuvant chemotherapy was allowed, if ended more than 6 months before enrolment. Main exclusion criteria were previous palliative chemotherapy for metastatic disease; previous chemotherapy including irinotecan or oxaliplatin; symptomatic cardiac disease or myocardial infarction in the past 24 months or uncontrolled arrhythmia; active infections; inflammatory bowel disease; total colectomy. The study was conducted in accordance to the Helsinki declaration and to the Good Clinical Practice guidelines. Patients provided their written informed consent before registration. The protocol was approved by the ethics committees of all participating institutions.

### Treatment

**Figure 1:**
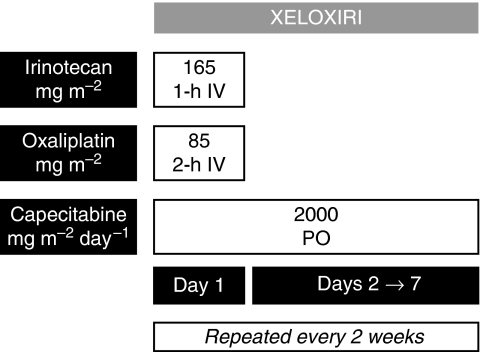
XELOXIRI regimen: treatment schedule. IV, intravenous; PO, per os

The chemotherapy regimen consisted of irinotecan 165 mg m^−2^ i.v. in 250 ml of NaCl 0.9% over 1 h, followed immediately by oxaliplatin 85 mg m^−2^ i.v. in 250 ml dextrose 5%, as used in the FOLFOXIRI regimen. Capecitabine was administered at the dose of 2000 mg m^−2^ per day orally in two divided doses from day 1 to day 7 (Figure 1). Treatment was repeated every 2 weeks and administered until evidence of disease progression, unacceptable toxicity, patient refusal or for a maximum of 12 cycles. Toxicities were graded according to the National Cancer Institute Common Terminology Criteria (NCI CTC) version 3.0. Treatment was delayed until recovery in case of neutrophils <1.000 per mm^3^, platelets <100.000 per mm^3^ or diarrhoea or stomatitis grade >1 on the planned day of treatment. In the case of peripheral neurotoxicity grade >2, oxaliplatin was interrupted. In the case of previous dose-limiting toxic effects, treatment was continued after resolution of the event with doses of oxaliplatin, irinotecan and capecitabine reduced by 25%, except in the case of grade 3–4 diarrhoea, when only irinotecan and capecitabine doses were reduced by 25%. In the case of life-threatening toxic effects, treatment was definitively interrupted or continued at doses reduced by 50%. To prevent nausea and vomiting, 5-hydroxytryptamine-3 receptor antagonists + dexamethasone 16 mg were administered i.v. before chemotherapy, and repeated as i.m. injections at standard doses in the 2 following days. Atropine 0.25 mg subcutaneously was given to treat cholinergic syndrome, and repeated as prophylaxis of future events in the following cycles. Loperamide 2 mg, orally every 2 h, and oral rehydration were prescribed in case of delayed diarrhoea. No prophylactic treatment with white blood cell growth factors for neutropenia was recommended.

### Evaluation criteria

Pretreatment evaluation included medical history and physical examination, ECOG PS assessment, complete blood cell counts with differential, complete blood profile, carcinoembryonic antigen (CEA), electrocardiogram, chest and abdominal tomography (CT) scan and any other appropriate diagnostic procedure to evaluate metastatic sites. During treatment, a physical examination and a complete blood cell count, AST, ALT, total bilirubin and creatinine were performed every 2 weeks. Evaluation of tumour response was performed with CT scan every 8 weeks according to the standard RECIST criteria (Therasse *et al*, 2000). The best overall response for each patient was reported. All results were reviewed by an independent radiologist and had to be confirmed 28 days or more after initial documentation of the response. The overall response rate was calculated according to the intention-to-treat analysis. Progression-free survival was calculated from the day of registration to the date of first observation of clinical and/or radiological evidence of progression or death, whichever occurred first. Overall survival (OS) was calculated from the day of registration to the date of death or last contact. OS and PFS were estimated using the Kaplan–Meier method.

### Statistical analysis

The minimax two-stage sequential design described by Simon (1989) was used to determine the number of patients to be included. As responses with standard reference combinations of irinotecan + capecitabine or oxaliplatin + capecitabine are observed in about 40–50% of patients, a response rate ⩾70% for a new regimen that has acceptable toxic effects would be considered promising. Therefore, the design parameters p0 (response rate in null hypothesis) and p1 (response rate in alternative hypothesis) selected were 0.50 and 0.70, respectively. Also considering an *α* and *β* error probability of 0.05 and 0.20, respectively, the first stage of the study required 23 patients, and if at least 13 objective responses were observed, 13 additional patients had to be enrolled in the second stage of the study. The regimen was considered interesting for further investigation if ⩾24 objective responses out of 36 evaluable patients were observed.

## Results

### Patient characteristics

From February 2006 through March 2007, a total of 36 patients with unresectable metastatic colorectal cancer were enrolled. The baseline patients characteristics are reported in Table 1. Median age was 65 years (range 42–73); 24 patients (67%) had synchronous metastases at diagnosis, half of patients had multiple sites of metastases, 44% had liver involvement ⩾25 and 25% had received adjuvant treatment before the enrolment into the study.

### Toxicity and dose administration

All patients were assessable for safety. A total of 342 cycles of chemotherapy were administered with a median of 12 cycles per patient (range 2–12). Overall, the treatment was relatively well tolerated without frequent grade 3–4 toxicities (Table 2), except for neutropenia and diarrhoea. Indeed, 8 patients (22%) experienced at least one episode of grade 3 diarrhoea and 3 (8%) of grade 4. Neutropenia of grade 3–4 was observed in 30% of patients, with febrile neutropenia in 4 cases (11%). Other grade 3–4 toxicities included nausea in 3% of patients, vomiting in 6%, thrombocytopenia in 8% and peripheral neurotoxicity in 6%. Three patients were hospitalised for febrile neutropenia and diarrhoea, and one patient died because of sepsis. One hundred and forty-three cycles (43%) were administered with a dose reduction of at least one drug. Most cycles (82%) were administered every 2 weeks as per protocol, whereas 61 cycles (18%) were delayed, usually because of toxicity. The median dose intensities of irinotecan, oxaliplatin and capecitabine calculated during the entire period of treatment among the 36 patients were 63 mg m^−2^ per week (76% of planned), 36 mg m^−2^ per week (85% of planned) and 4842 mg m^−2^ per week (69% of planned), respectively. Although the use of G-CSF was not planned, it was used in five (1.5%) cycles.

### Antitumour activity and survival

At an intention-to-treat analysis, we observed 22 (61%) partial and 2 (6%) complete responses, for an overall response rate of 67% (95% confidence interval 51.4–82%). In addition, nine patients (25%) achieved a disease stabilisation as best response and only three patients (8%) progressed. Eight patients underwent surgical removal and/or radiofrequency ablation of residual metastases after response to chemotherapy with a radical (R0) resection performed in 6 (17%) of the 36 initially unresectable patients (38% among patients with liver involvement only).

**Figure 2:**
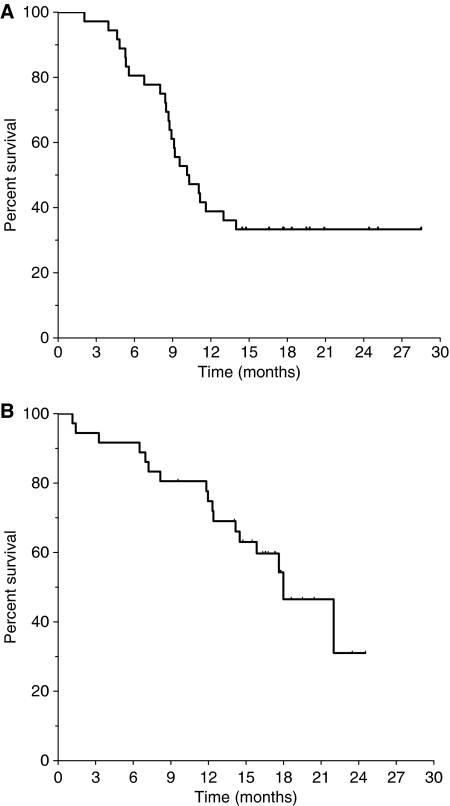
Kaplan–Meier estimates of progression-free survival (**A**) and overall survival (**B**). Panel **A** represents the progression free survival: median 10.1 mos; 95% CI 7.4–12.8. Panel **B** represents the overall survival: median 17.9 mos; 95% CI 13.5–22.5

After a median follow-up of 17.7 months, with 24 patients who experienced disease progression and 17 who died, the median progression-free survival and overall survival were 10.1 (95% confidence interval 7.4-12-8) and 17.9 (95% confidence interval 13.5–22.5) months, respectively (Figure 2A and B). Twenty-one patients received a second-line treatment after disease progression, in most cases containing cetuximab.

## Discussion

In the past years, improvements in chemotherapy for the treatment of metastatic colorectal cancer have resulted in significant benefits in terms of antitumour activity and efficacy (Punt, 2004; Saunders and Iveson, 2006). Several studies have suggested that the best results can be achieved exposing patients to all three main active cytotoxics (5-FU, irinotecan and oxaliplatin) (Grothey *et al*, 2004; Folprecht *et al*, 2005). In particular, a phase III study conducted by the GONO demonstrated the superiority of the first-line triplet FOLFOXIRI *vs* a standard doublet in terms of activity and efficacy. However, 5-FU had to be administered as a 48-h continuous infusion by a central venous catheter to make the combination feasible (Falcone *et al*, 2002, 2007; Masi *et al*, 2004).

Our report is the first multicenter phase II study evaluating the activity of a first-line triplet combination of irinotecan and oxaliplatin associated with capecitabine instead of 5-fluorouracil in the treatment of metastatic colorectal cancer. The overall response rate of 67% and the median PFS of 10.1 months are comparable to those obtained in phases II and III trials with FOLFOXIRI. Moreover, our results are also comparable to those of a single-centre phase I–II study recently reported by the ITMO with the combination of irinotecan 180 mg m^−2^ on day 1, oxaliplatin 85 mg m^−2^ on day 2 and capecitabine 2000 mg m^−2^ per day from day 2 to day 6 (COI), in 29 mCRC patients (Bajetta *et al*, 2007). However, the ITMO COI schedule seems more complex with a lengthy outpatient 2-day schedule for irinotecan and oxaliplatin administration.

The major concern with the XELOXIRI regimen is the gastrointestinal toxicity, in particular in terms of grade 3–4 diarrhoea that was experienced by 30% of patients. The incidence of severe diarrhoea is apparently higher to that observed in studies with FOLFOXIRI and also to that reported by Bajetta *et al* with the COI regimen, in this case probably because of the lower dose intensity of capecitabine administered. Also, two recent phase III trials comparing the combination of irinotecan with 5-fluorouracil or capecitabine (at the dose of 2000 mg m^−2^ per day on days 1–14 every 21 days) reported a high (about 40%) rate of grade 3–4 diarrhoea with capecitabine and irinotecan (Fuchs *et al*, 2007; Kohne *et al*, 2008). The incidence of diarrhoea with this combination may be reduced by slightly lowering the dose of the two drugs, without impairing the activity (Punt and Koopman, 2008; Reinacher-Schick *et al*, 2008). Finally, neutropenia was observed frequently in our study with at least one grade 3–4 episode in 30% of patients, but it was usually short lasting and rarely complicated, and it did not differ from that obtained with the infusional 5-FU triplet.

In conclusion, the substitution of capecitabine for infusional 5-fluorouracil, in combination with irinotecan and oxaliplatin, retained an interesting activity in the first-line treatment of metastatic colorectal cancer and could replace the need for an implanted central venous catheter. However, the incidence of grade 3–4 diarrhoea experienced with the XELOXIRI regimen seems higher than that with FOLFOXIRI and the regimen with the oral fluoropyrimidine seemed less manageable than that with infusional 5-fluorouracil. Therefore, a triple-drug combination of CPT-11 and L-OHP with capecitabine instead of infusional 5-FU as we used is not a preferable alternative to FOLFOXIRI, but can be considered for patients with mCRC refusing or with contraindications to the implantation of a central venous catheter.

## Figures and Tables

**Table 1:** Patients characteristics

**Characteristic**	**Number of patients (%)**
Patients	36
Median age (years; range)	65 (42–73)
	
*Sex*	
Male	28 (78)
Female	8 (22)
	
*ECOG PS*	
0	32 (89)
1	3 (8)
2	1 (3)
	
*Primary tumour site*	
Colon	26 (72)
Rectum	10 (28)
	
Previous surgery on primary tumour	29 (81)
Previous adjuvant chemotherapy	9 (25)
Previous radiotherapy	2 (6)
	
*Number of metastatic sites*	
Single	18 (50)
Multiple	18 (50)
	
*Timing of metastases*	
Synchronous	24 (67)
Metachronous	12 (33)
	
*Sites of disease*	
Liver	29 (81)
Lung	13 (36)
Lymph nodes	10 (28)
Peritoneum	6 (17)
Other	3 (8)
	
*Liver involvement*	
<25%	20 (56)
25–50%	7 (19)
>50%	9 (25)

**Table 2:** Maximum toxicities per patient (36 patients)

	**National cancer institute common terminology criteria grade (%)**			
**Adverse event**	**1**	**2**	**3**	**4**
Neutropenia^a^	11	28	14	16
Thrombocytopenia	33	6	8	0
Anaemia	56	19	0	0
Nausea	44	31	3	0
Vomiting	14	17	6	0
Diarrhoea	31	25	22	8
Stomatitis	17	17	0	0
Peripheral Neurotoxicity	42	17	6	0
Fatigue	19	28	3	0
Palmar-plantar erythrodysaesthesia	6	3	0	0

a Febrile neutropenia in 11% of patients.
